# Preventive effects of nucleoprotein supplementation combined with intermittent loading on capillary regression induced by hindlimb unloading in rat soleus muscle

**DOI:** 10.14814/phy2.13134

**Published:** 2017-02-27

**Authors:** Yusuke Hirayama, Ryosuke Nakanishi, Noriaki Maeshige, Hidemi Fujino

**Affiliations:** ^1^ Department of Rehabilitation ScienceKobe University Graduate School of Health SciencesKobeJapan

**Keywords:** Capillary regression, intermittent loading, nucleoprotein, skeletal muscle

## Abstract

Physical inactivity leads to muscle atrophy and capillary regression in the skeletal muscle. Intermittent loading during hindlimb unloading attenuates the muscle atrophy, meanwhile the capillary regression in the skeletal muscle is not suppressed. Nucleoprotein has antioxidant capacity and may prevent capillary regression. Therefore, we assessed the combined effects of intermittent loading with nucleoprotein supplementation on capillary regression induced by hindlimb unloading. Five groups of rats were assigned: control (CON), 7 days hindlimb unloading (HU), HU plus nucleoprotein supplementation (HU + NP), intermittent loading during HU (HU + IL), and intermittent loading combined with nucleoprotein supplementation during HU (HU + IL + NP). Seven days HU resulted in decrease in capillary number‐to‐fiber number (C/F) ratio accompanied with disuse‐associated changes in fetal liver kinase‐1 (Flk‐1), a proangiogenesis factor, and thrombospondin‐1 (TSP‐1), an antiangiogenesis factor, in the soleus muscle. In addition, citrate synthase (CS) activity was decreased and protein level of superoxide dismutase (SOD)‐2 was increased. Neither nucleoprotein supplementation nor intermittent loading prevented the decrease in the C/F ratio, whereas nucleoprotein supplementation combined with intermittent loading prevented the regression of capillary during unloading. Moreover, the levels of Flk‐1, TSP‐1, and SOD‐2 protein and the CS activity were maintained up to control levels. These results suggested that nucleoprotein supplementation combined with intermittent loading was effective to prevent capillary regression induced by muscle atrophy.

## Introduction

Capillaries play a critical role in deliver of oxygen and nutrients to skeletal muscle, and have remarkable plasticity (Olfert and Birot 2011). It is well established that the decrease in physical activity leads to capillary regression (Kano et al. 2000; Fujino et al. 2005; Roudier et al. 2010; Olfert and Birot 2011). The capillary regression in skeletal muscle is most likely to drop fatigue resistance, since the capillary related to exercise capacity (Tadaishi et al. 2011). Indeed, the shortening of treadmill running time consisted with capillary regression in rat soleus muscle has been demonstrated (Tanaka et al. 2015). Therefore, it is necessary to prevent the capillary regression under disuse condition.

The capillary regression induced by hindlimb unloading (HU) is regulated by the imbalance between pro‐ and anti‐angiogenic signals: a decrease in fetal liver kinase‐1 (Flk‐1) and an increase in thrombospondin‐1 (TSP‐1) (Roudier et al. 2010). It has been considered that these changes are resulted from the decrease in demand for oxygen (Fujino et al. 2005; Egginton 2010; Olfert and Birot 2011) and increase in oxidative stress (Kanazashi et al. 2013, 2014). The oxygen demand is correlated with oxidative enzyme activity (Blomstrand et al. 2011), and then the relationship between citrate synthase (CS) activity and capillary‐to‐fiber (C/F) ratio has been shown (Poole and Mathieu‐Costello 1996). In addition, some reports have demonstrated that HU decreases CS activity in skeletal muscle (Stump et al. 1997; Cassano et al. 2010; Wagatsuma et al. 2011). Meanwhile, the oxidative stress is induced by reactive oxygen species (ROS) overproduction from mitochondria (Min et al. 2011; Powers et al. 2012; Powers 2014), which is reflected to the upregulation of superoxide dismutase (SOD) protein expression in unloaded muscle as compensation (Zelko et al. 2002; Siu and Alway 2005; Kanazashi et al. 2013, 2014). Thus, it is necessary to maintain the oxidative enzyme activities and to suppress oxidative stress for preventing the capillary regression induced by inactivity.

Intermittent loading attenuates muscle wasting induced by HU (Dupont‐Versteegden et al. 2002; Miyazaki et al. 2008), meanwhile it does not suppress the capillary regression in skeletal muscle (Kanazashi et al. 2014). Therefore, an additional intervention is needed such as nutritional support for preventing not only atrophy but also capillary regression. In previous study, we have shown that an antioxidant supplementation is effective to prevent both of capillary regression and mitochondrial dysfunction induced by HU (Kanazashi et al. 2013, 2014). Nucleoprotein may also have antioxidant capacity since dietary nucleotide, a component of nucleoprotein, has been shown the activating antioxidant enzymes in serum of rat (Xu et al. 2013).

We hypothesized that nucleoprotein supplementation combined with intermittent loading would prevent not only muscle wasting but also capillary regression induced by HU. For the investigation, we have set up the HU‐duration in 7 days since dynamic changes in capillary and molecular are occurred in the first week of HU (Kano et al. 2000; Roudier et al. 2010).

## Materials and Methods

### Experimental groups

Thirty‐five adult male Sprague–Dawley rats were used in this study. Rats aged 8 weeks (294.5 ± 1.5 g) were purchased from Japan SLC (Hamamatsu, Japan). After 1 week acclimatization, rats were divided into following five groups: control (CON, *n* = 7), 7 days HU (HU, *n* = 7), nucleoprotein supplementation during HU (HU + NP, *n* = 7), intermittent loading during HU (HU + IL, *n* = 7), and nucleoprotein supplementation combined with intermittent loading during HU (HU + IL + NP, *n* = 7). The rats in HU + NP and HU + IL + NP groups were orally administrated nucleoprotein (800 mg/kg/day, Nissan chemical industries, Tokyo, Japan) dissolved 5% in gum arabic solution with catheter twice in a day. The rats in the other groups were orally administrated the same volume of 5% gum arabic solution. The gum arabic was used as an emulsifier. After 1‐week administration for acclimatizing, the rats in HU, HU + NP, HU + IL, and HU + IL + NP groups had been unloaded for 1 week. All rats were administrated gum arabic solution or nucleoprotein during HU. The rats were housed in an isolated and environmental controlled room at 22 ± 2°C on a 12–12 h light–dark cycle, and were fed food and drinking water ad libitum. This study was approved by the Institutional Animal Care and Use Committee, and was performed according to the Kobe University Animal Experimentation Regulations. All experiments were conducted in accordance with the National Institutes of Health (NIH) Guidelines for the Care and Use of Laboratory Animals (National Research Council 1996).

### Hindlimb unloading and intermittent loading

Hindlimb unloading was carried out according to previously described procedure (Morey et al. 1979) with modify. In brief, every rat was suspended with tail by an adhesive tape and a kite string, and prevented its hindlimb from bearing on a floor and sides of a cage. The forelimbs were allowed to maintain contact on the floor of the cage. The intermittent loading was carried out by releasing from unloading for an hour a day at night.

### Sample preparation

About twelve hours after from final intermittent loading, all rats were deeply anesthetized by pentobarbital (50 mg/kg, *i.p*.). Blood samples for assessment of plasma antioxidant enzyme activity were collected from abdominal portion of vena cava. The blood samples were centrifuged at 1200 *g* for 10 minutes and the obtained plasma were stored −80°C until analysis. Thereafter, soleus muscles were removed and weighed. The soleus muscles were then frozen immediately in an acetone/dry ice bath and stored −80°C until analysis.

### Histological analysis

Transverse soleus muscle sections of 12‐μm thickness were cut on a cryostat (CM‐1510S, Leica Microsystems, Mannheim, Germany) at −25°C and were mounted on glass slides. The sections were stained for myofibrillar adenosine triphosphatase (ATPase) following preincubation at pH 4.2 and alkaline phosphatase to identify muscle fiber type and capillary, respectively. The stained sections were used for measuring fiber cross‐sectional area (FCSA), slow fiber composition, and counting the number of capillary and muscle fiber to calculate capillary‐to‐fiber (C/F) ratio with Image J software program (NIH, Bethesda, MD).

### SDS polyacrylamide gel electrophoresis and western blot analysis

The muscles were homogenized on ice in homogenization buffer (50 mmol/L Tris‐HCl pH 8.0, 120 mmol/L NaCl, 1% NP‐40, 20 mmol/L NaF, 1 mmol/L EDTA, 1 mmol/L EGTA, 15 mmol/L sodium pyrophosphate, 30 mmol/L *β*‐glycerophosphate, 2 mmol/L Na_2_VO_4_, and 1% protease inhibitor cocktail). The homogenates were centrifuged at 1700 *g* for 10 min at 4°C and supernatants were collected. Total protein concentration of the supernatants was determined as above. The supernatants were solubilized in sample loading buffer (50 mmol/L Tris–HCl pH 6.8, 2% sodium dodecyl sulfate, 10% glycerol, 5% 2‐mercaptoethanol, and 0.005% bromophenol blue) and boiled for 10 min at 80°C.

The proteins (30 μg/lane) were separated by SDS polyacrylamide gel electrophoresis (SDS‐PAGE) and transferred onto polyvinylidene fluoride (PVDF) membranes. The membranes were blocked for 1 h in 5% skimmed milk in phosphate‐buffered saline with Tween‐20 (PBST). After the blocking, the membranes were incubated with primary antibodies; anti‐VEGF (1:200 in PBST, sc‐7269; Santa cruz biochemistry Dallas, TX), anti‐Flk‐1 (1:100 in PBST sc‐504; Santa cruz biochemistry) anti‐TSP‐1 (1:100 in PBST, sc‐59887; Santa cruz biochemistry), anti‐SOD‐2 (1:200 in PBST, #13141; Cell Signaling Technology Japan, Tokyo, Japan), or anti‐*β*‐actin (1:1000 in PBST, sc‐47778; Santa cruz biochemistry, CA). The primary antibodies were detected by anti‐rabbit or anti‐mouse IgG conjugated to horseradish peroxidase (GE Healthcare, Waukesha, WI), and visualized by a chemiluminescent reagent (Ez West Lumi, ATTO, Tokyo, Japan) and determined with an image reader (LAS‐1000, Fujifilm, Tokyo, Japan).

### Biochemical analysis

Citrate synthase activity of the soleus muscle was determined according to previously described procedure (Srere 1963). In brief, the muscle were homogenized on ice in homogenization buffer containing 50 mmol/L Tris‐HCl pH 8.0, 50 mmol/L NaCl, 1 mmol/L EDTA, 1% protease inhibitor cocktail. The homogenates were centrifuged at 1,700 *g* for 10 min at 4°C and supernatants were collected. The supernatants were solubilized in reaction buffer containing 0.1 mmol/L DTNB and 0.3 mmol/L acetyl‐CoA. The reaction was initiated by oxaloacetic acid (0.5 mmol/L final concentration). Absorbance at 412 nm was measured with a spectrophotometer for 5 min. The values were normalized with the total protein concentration of the supernatants.

Superoxide dismutase activity in plasma was measured using the superoxide dismutase assay kit (435‐70601; Wako, Tokyo, Japan). Briefly, superoxide anion generated by xanthine and xanthine oxidase reaction produces diformazan with the maximal absorbance at 560 nm. The SOD activity was measured through the inhibition rate of the diformazan production.

### Statistical analysis

All data are presented as means ± SEM. The differences were assessed by one‐way ANOVA followed by Tukey–Kramer's post hoc test to determine specific group differences. For all tests, the level of significance was set at *P *<* *0.05.

## Results

### Body mass, absolute, relative soleus mass, fiber cross‐sectional area, and slow fiber composition

Mean body mass, absolute and relative soleus muscle mass, and FCSA were markedly decreased due to 7 days HU (Table 1). The loss of absolute soleus muscle mass was attenuated by only combined treatment (HU + IL + NP). Meanwhile the loss of FCSA was attenuated by intermittent loading (HU + IL and HU + IL + NP). There was no transformation of slow fiber composition.

**Table 1:** Body, muscle and relative muscle‐to‐body mass, fiber cross‐sectional area, and slow fiber composition

	Body mass (g)	Soleus mass (mg)	Relative soleus mass (mg/100 g)	Fiber cross‐sectional area (μm^2^)	Slow fiber composition (%)
CON	345 ± 10	153 ± 5	44 ± 1	3273 ± 115	87 ± 1
HU	297 ± 7*	96 ± 2*	32 ± 1*	1707 ± 53*	82 ± 2
HU + NP	288 ± 4*	95 ± 2*	33 ± 1*	1751 ± 32*	83 ± 2
HU + IL	304 ± 7*	106 ± 2*	35 ± 1*	2144 ± 62*, †, ‡	85 ± 2
HU + IL + NP	308 ± 5*	108 ± 2*, †, ‡	35 ± 1*	2264 ± 102*, †, ‡	84 ± 2

Values are mean ± SEM (*n* = 7). CON, control; HU, hindlimb unloading; NP, nucleoprotein supplementation; IL, intermittent loading. *, ^†^ and ^‡^ indicate significant differences from CON, HU, and HU + NP groups, respectively (*P* < 0.05). One‐way ANOVA followed by Tukey–Kramer's post hoc test was used for statistical analysis.

### Number of capillary

**Figure 1:**
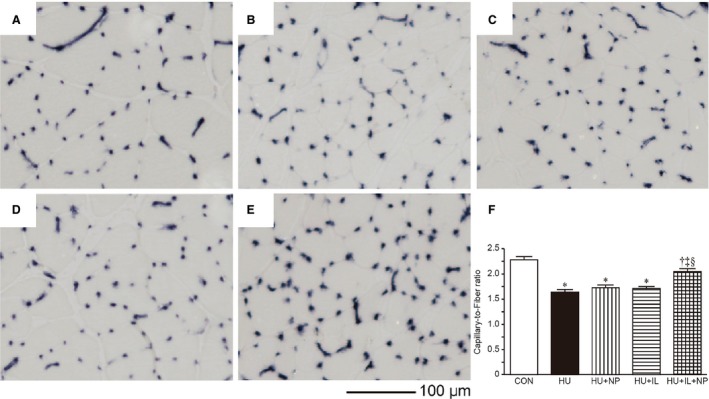
Transverse sections stained for alkaline phosphatase (A–E) and C/F ratio (F) of the soleus muscles. CON (A), HU (B), HU + NP (C), HU + IL (D), and HU + IL + NP (E). Capillaries were visualized as black dots. Bar = 100 μm. Values are presented as the means ± SEM (*n* = 7). CON, control; HU, hindlimb unloading; NP, nucleoprotein supplementation; IL, intermittent loading. *, ^†^, ^‡^ and ^§^ indicate significant differences from CON, HU, HU + NP, and HU + IL groups, respectively (*P* < 0.05). One‐way ANOVA followed by Tukey–Kramer's post hoc test was used for statistical analysis.

A representative image of the stained capillary from soleus muscles is shown in Figure 1A–E. The C/F ratios were significantly decreased by 7 days HU. The decrease in C/F ratio was prevented by the combined treatment (HU + IL + NP) although other treatments (HU + NP and HU + IL) did not prevent the decrease in C/F ratio (Fig. 1F).

### Pro and antiangiogenic factors

**Figure 2:**
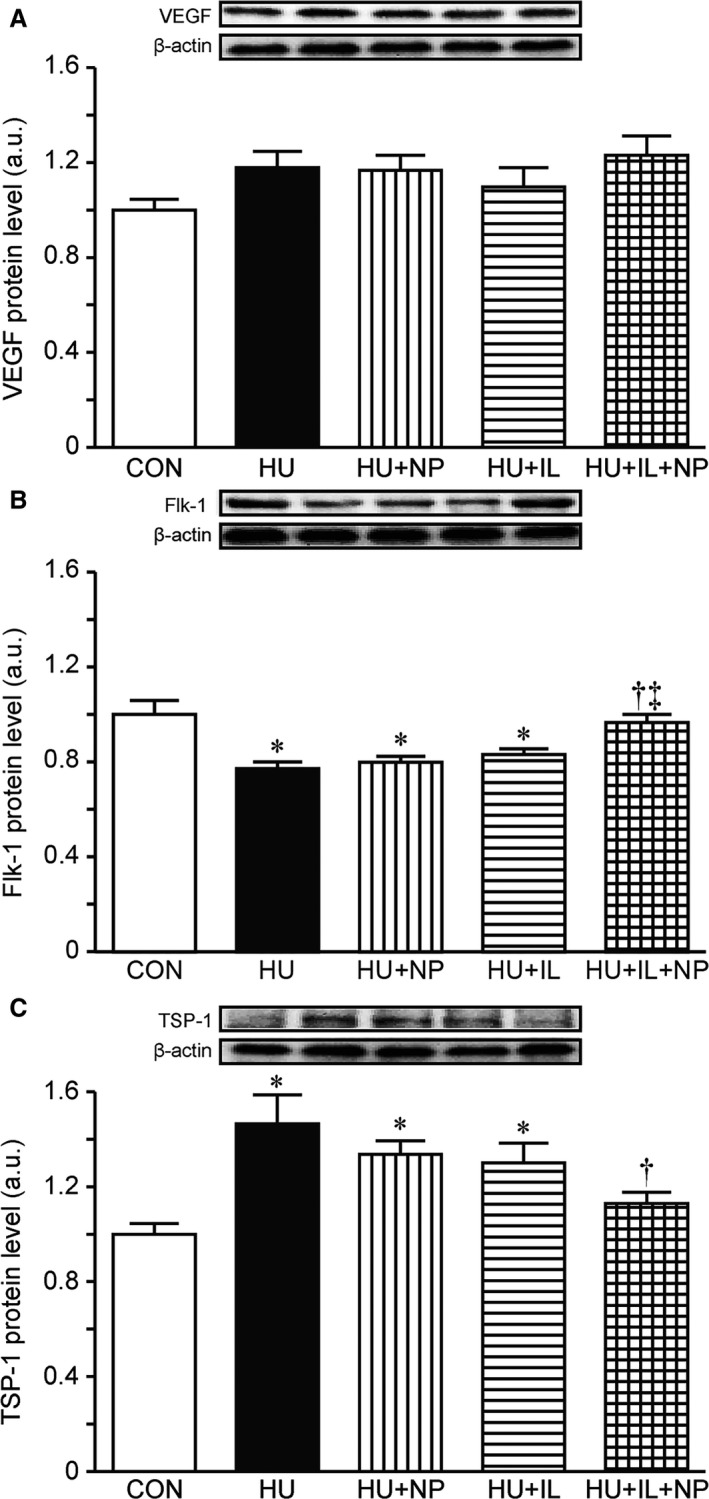
Protein levels of VEGF (A), Flk‐1 (B), and TSP‐1 (C) in the soleus muscles. Values are presented as means ± SEM (*n* = 7). CON, control; HU, hindlimb unloading; NP, nucleoprotein supplementation; IL, intermittent loading. *, ^†^ and ^‡^ indicate significant differences from CON, HU, and HU + NP, respectively (*P* < 0.05). One‐way ANOVA followed by Tukey–Kramer's post hoc test was used for statistical analysis.

Protein contents of VEGF, Flk‐1, and TSP‐1 were analyzed by SDS‐PAGE and western blot. There was no significant change in VEGF protein levels (Fig. 2A). Meanwhile, protein level of Flk‐1; proangiogenic factor was significantly decreased (Fig. 2B), that of TSP‐1; antiangiogenic factor was significantly increased (Fig. 2C) due to 7 days HU. Although nucleoprotein supplementation (HU + NP) or intermittent loading (HU + IL) treatment did not suppress these HU‐related changes, the combined treatment (HU + IL + NP) suppressed.

### Oxidative enzyme activity

**Figure 3:**
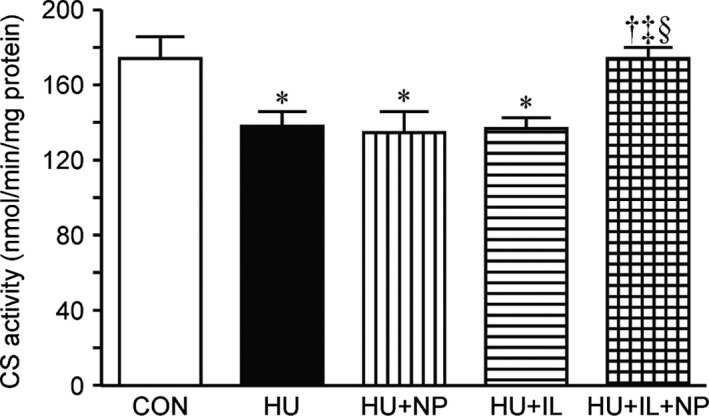
Citrate synthase (CS) activity in the soleus muscles. Values are presented as the means ± SEM (*n* = 7). CON, control; HU, hindlimb unloading; NP, nucleoprotein supplementation; IL, intermittent loading. *, ^†^, ^‡^ and § indicate significant differences from CON, HU, HU + NP, and HU + IL groups, respectively (*P* < 0.05). One‐way ANOVA followed by Tukey–Kramer's post hoc test was used for statistical analysis.

Citrate synthase activity was analyzed by calculating reaction rate normalized protein level. The CS activity was markedly decreased due to 7 days HU. Although single treatment (HU + NP or HU + IL) did not prevent this reduction, the combined treatment (HU + IL + NP) prevented (Fig. 3).

### Oxidative stress level

**Figure 4:**
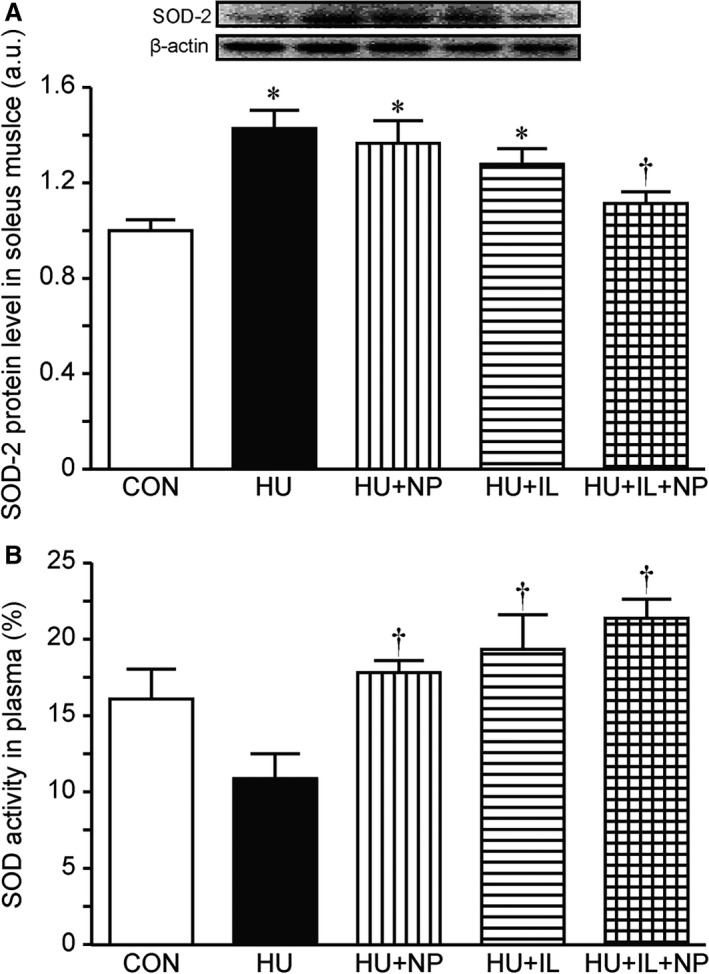
Protein levels of SOD‐2 in the soleus muscles (A) and activity of SOD in plasma (B). Values are presented as the means ± SEM (*n* = 7). CON, control; HU, hindlimb unloading; NP, nucleoprotein supplementation; IL, intermittent loading. * and ^†^ indicate significant difference from CON and HU groups, respectively (*P* < 0.05). One‐way ANOVA followed by Tukey–Kramer's post hoc test was used for statistical analysis.

SOD‐2 protein levels in soleus muscle were measured as markers of oxidative stress by SDS‐PAGE and western blot. The protein levels of SOD‐2 were significantly increased by 7 days HU, however, the combined treatment (HU + IL + NP) suppressed the increase. Meanwhile single treatment (HU + NP or HU + IL) did not suppress the increase (Fig. 4A).

Superoxide dismutase activity tends to be decreased by 7 days HU. The all treatment (HU + NP, HU + IL, and HU + IL + NP) increased SOD activity compared to HU (Fig. 4B).

## Discussion

The novel finding of this study was that nucleoprotein supplementation combined with intermittent loading prevented capillary regression by suppressing oxidative stress during chronic unloading, nevertheless the single intervention of nucleoprotein supplementation did not prevent capillary regression and increase in oxidative stress.

Unloading for 1 week resulted in soleus muscle atrophy and capillary regression reflected by decrease in FCSA and C/F ratio, respectively, meanwhile intermittent loading attenuated the atrophy. Therefore, intermittent loading during unloading is considered to be effective for prevention of soleus muscle atrophy, and these results are congruent with the earlier studies (Dupont‐Versteegden et al. 2002; Miyazaki et al. 2008; Kanazashi et al. 2014). However, intermittent loading did not prevent capillary regression as well as the previous report (Kanazashi et al. 2014). This is likely to be due to overload since exercise associates the overproduction of free radicals in skeletal muscle (Polotow et al. 2014).

The combination of nucleoprotein supplementation and intermittent loading attenuated the capillary regression in this study. These results are caused by changes in pro and antiangiogenic factors; Flk‐1 and TSP‐1. Roudier et al. have reported that the capillary regression is occurred due to the decrease in Flk‐1 protein expression and the increase in TSP‐1 protein expression without a change in VEGF protein expression at the 7th day of HU (Roudier et al. 2010). The protein levels of Flk‐1 and TSP‐1 of HU + IL + NP group in this study were maintained at control level. These results indicate that the prevention of capillary regression by the combination of nucleoprotein supplementation and intermittent loading was likely to be resulted from the regulation of Flk‐1 and TSP‐1.

Angio‐adaptation is deeply coupled to intensity of oxidative metabolic profile, the composition of muscle fiber type, and the expression level of oxidative stress (Hudlicka et al. 1992; Kanazashi et al. 2014). Oxidative enzyme activity, *e.g*. CS activity, has positive correlation with C/F ratio in soleus muscle (Poole and Mathieu‐Costello 1996). In this study, the CS activity in HU, HU + NP, and HU + IL groups was lower, consistent with capillary regression. Since slow fiber composition was not changed, involvement of slow‐to‐fast isoforms transition was little in this study. Meanwhile, oxidative stress induces apoptosis in endothelial cells and lead to capillary regression (Kanazashi et al. 2013, 2014). The expression level, SOD protein is increased in unloaded muscle as compensation (Zelko et al. 2002; Siu and Alway 2005; Kanazashi et al. 2013, 2014). The higher expression levels of SOD‐2 protein, that means higher oxidative stress, in HU, HU + NP, HU + IL groups were also consistent with capillary regression. Whereas both CS activity and SOD‐2 protein level in HU + IL + NP group were kept at CON level. Therefore, the maintaining CS activity and the suppressing oxidative stress may contribute to preventing capillary regression through pro‐ and antiangiogenic factors.

Nucleoprotein supplementation without intermittent loading resulted in an increase in the SOD activity in plasma. This change was congruent with Xu's report that showed antioxidative effects of nucleotide supplementation (Xu et al. 2013), and suggested that nucleoprotein also has antioxidant capacity as well as nucleotide. However, the additional effect of the combination was not observed. Although exercise also increases the SOD activity in plasma (Cardoso et al. 2013; Peeri et al. 2013), exercise produces the oxidative stress in skeletal muscle (Polotow et al. 2014). Thus, intermittent loading might attenuate the effect of nucleoprotein on SOD activity especially in plasma.

Although nucleoprotein may have antioxidant capacity, nucleoprotein supplementation did not prevent the decrease in CS activity and the increase in SOD‐2 protein expression. These results are not consistent with our previous study that nucleoprotein supplementation attenuates capillary regression and decrease in oxidative enzyme activity (Kanazawa et al. 2013) The difference between the present and the previous study was duration of unloading. The changes in capillary and angiogenic factors are markedly in the first week of unloading (Kano et al. 2000; Roudier et al. 2010). These serious changes may be caused by oxidative stress. Unloading for 7 days increased the level of carbonyl protein content, a marker of oxidative stress (Vitadello et al. 2014), whereas unloading for 14 days did not increase the carbonylation (Dupre‐Aucouturier et al. 2015). In addition, Liu et al. have demonstrated that oxidative stress induced by hindlimb immobilization peak at 7th day through 21 days experiment (Liu et al. 2003). Therefore, the prevention of capillary regression in this study might depend on oxidative stress level. The significant slow‐to‐fast fiber transition was not observed in this study. Thus, the prevention of capillary regression in this study was likely to depend on oxidative stress level, but not fiber transition at least the first week of unloading. Furthermore, our results were not also consistent with other previous studies that antioxidant supplementation suppresses oxidative stress (Kanazashi et al. 2013, 2014). Powers has reviewed that the oxidative stress is mainly derived from mitochondria in inactivated muscle (Powers 2014). In addition, Fernstrom et al. have demonstrated that nucleotides are promoted to transport into mitochondrial matrix by muscle activity (Fernstrom et al. 2004). These review and report suggest that nucleoprotein supplementation has few effects on oxidative stress in skeletal muscle under disuse condition. Therefore, the combination with intermittent loading was required to transport nucleotide into mitochondrial matrix for suppressing oxidative stress.

A limitation of this study is that we determined only SOD‐2 protein level to assess oxidative stress. We were specifically interested in effects of nucleoprotein supplementation and intermittent loading on capillary regression. Future studies, however, should be performed to analyze oxidative stress.

## Conclusion

Nucleoprotein supplementation combined with intermittent loading was effective to prevent capillary regression induced by muscle atrophy. This study indicates the importance of nutritional and physical strategy for treatment during disuse.

## Conflict of Interest

There was no conflict of interest to disclose.
